# Age-adjusted quick Sequential Organ Failure Assessment score for predicting mortality and disease severity in children with infection: a systematic review and meta-analysis

**DOI:** 10.1038/s41598-021-01271-w

**Published:** 2021-11-04

**Authors:** Sohyun Eun, Haemin Kim, Ha Yan Kim, Myeongjee Lee, Go Eun Bae, Heoungjin Kim, Chung Mo Koo, Moon Kyu Kim, Seo Hee Yoon

**Affiliations:** 1 Department of Pediatrics, Severance Children’s Hospital, Yonsei University College of Medicine, Seoul, South Korea; 2 Biostatistics Collaboration Unit, Department of Biomedical Systems Informatics, Yonsei University College of Medicine, Seoul, South Korea; 3 Department of Emergency Medicine, Yonsei University College of Medicine, Seoul, South Korea

**Keywords:** Infectious diseases, Predictive markers, Paediatrics

## Abstract

We assessed the diagnostic accuracy of the age-adjusted quick Sequential Organ Failure Assessment score (qSOFA) for predicting mortality and disease severity in pediatric patients with suspected or confirmed infection. We conducted a systematic search of PubMed, EMBASE, the Cochrane Library, and Web of Science. Eleven studies with a total of 172,569 patients were included in the meta-analysis. The pooled sensitivity, specificity, and diagnostic odds ratio of the age-adjusted qSOFA for predicting mortality and disease severity were 0.69 (95% confidence interval [CI] 0.53–0.81), 0.71 (95% CI 0.36–0.91), and 6.57 (95% CI 4.46–9.67), respectively. The area under the summary receiver-operating characteristic curve was 0.733. The pooled sensitivity and specificity for predicting mortality were 0.73 (95% CI 0.66–0.79) and 0.63 (95% CI 0.21–0.92), respectively. The pooled sensitivity and specificity for predicting disease severity were 0.73 (95% CI 0.21–0.97) and 0.72 (95% CI 0.11–0.98), respectively. The performance of the age-adjusted qSOFA for predicting mortality and disease severity was better in emergency department patients than in intensive care unit patients. The age-adjusted qSOFA has moderate predictive power and can help in rapidly identifying at-risk children, but its utility may be limited by its insufficient sensitivity.

## Introduction

Recently, remarkable progress has been achieved in reducing overall incidence of infectious diseases^1,2^. However, infectious diseases remain a leading cause of childhood morbidity and mortality globally^3,4^. Specifically, mortality of up to 20% has been reported in children with sepsis^5^, largely because the lack of specific signs and symptoms in children makes early diagnosis and treatment challenging^6^. In addition, rapid deterioration can occur when compensation fails in pediatric patients^7,8^. Thus, early recognition of sepsis is crucial to ensure timely management and to reduce mortality^9,10^.

Pediatric sepsis has been defined by the International Pediatric Sepsis Consensus Conference (IPSCC) as the presence of systemic inflammatory response syndrome (SIRS) with suspected or proven infection in a child^11^. However, the SIRS criteria lack specificity for identifying infected patients at high risk of mortality because children with non-infectious conditions can also meet the SIRS criteria; moreover, children who meet the SIRS criteria for sepsis often do not have organ dysfunction^12,13^.

In the case of adults, the Sepsis-1 and Sepsis-2 consensus definitions also adopted the SIRS criteria^14,15^. However, the Third International Consensus Definitions for Sepsis and Septic Shock (SEPSIS-3) task force revised the sepsis definition as the presence of a dysregulated host response that manifests as detrimental organ dysfunction, thus enabling the differentiation of sepsis from uncomplicated infections or non-infectious conditions^16,17^. Organ dysfunction is specifically defined by acute changes in the Sequential Organ Failure Assessment score (SOFA) with ≥ 2 points^16,17^.

The SEPSIS-3 task force also recommended the bedside tool, the quick SOFA (qSOFA), for the early recognition of adult patients with suspected infection at risk for poor outcomes. The qSOFA is a simplified version of the SOFA, comprising only three components: low systolic blood pressure (≤ 100 mmHg), high respiratory rate (≥ 22 breaths/min), and altered mental status (Glasgow Coma Scale [GCS] < 15)^16,17^. The SOFA and qSOFA showed better prognostic performance than the former sepsis criteria among adult patients in various clinical settings^17–20^. However, the SEPSIS-3 criteria were developed and validated for adult patients and did not consider age-dependent physiologic variables^16^.

Recently, there have been attempts to adapt the SEPSIS-3 criteria for pediatric patients^21,22^. The age-adjusted or age-adapted qSOFA, which is adjusted according to age-specific cutoffs, has shown promising results among pediatric patients in emergency department (ED)^23,24^ and intensive care unit (ICU) settings^13,25^. However, there is no systematic review and meta-analysis reporting the predictive value of the age-adjusted qSOFA in children. Therefore, we aimed to evaluate the performance of the age-adjusted qSOFA in predicting outcomes, including mortality and disease severity, among pediatric patients with suspected or confirmed infection.

## Materials and methods

This systematic review and meta-analysis is reported according to the Preferred Reporting Items for Systematic reviews and Meta-Analyses statement (PRISMA)^26^ and is registered with PROSPERO (CRD42021232257).

### Study selection, eligibility, and data extraction

Two authors (SHY and HK) independently conducted literature searches of PubMed, EMBASE, the Cochrane Library, and Web of Science, without language or time restrictions, on January 6, 2021, with the aim of finding eligible studies assessing the performance of age-adjusted qSOFA in predicting mortality and/or disease severity in pediatric patients with suspected or confirmed infection. Various combinations of the following key words were used in the systematic search: “Quick Sequential Organ Failure Assessment,” “qSOFA,” “quick SOFA,” “q-SOFA,” “quick-SOFA,” and “pediatric,” “child,” “adolescent,” “infant,” and “neonate.”

Studies were eligible if they aimed to assess the performance of age-adjusted qSOFA to predict mortality or disease severity in pediatric patients (aged < 18 years) with suspected or confirmed infection. We used the following as indicators reflecting disease severity: admission or transfer to an ICU (including a critical care unit), development of severe sepsis^11^, or prolonged hospital stay (dependent on the authors’ definition, regardless of duration). If enrolled patients received a diagnostic code (e.g., International Classification of Diseases code) indicative of an infection or were diagnosed with sepsis/septic shock via consensus definition, we accepted them as patients with confirmed infection. In addition, if enrolled patients had signs or symptoms of infection (e.g., fever), or were treated for a bacterial infection (e.g., treated with therapeutic antibiotics), we inferred suspected infection. Studies were included if they reported sufficient data to construct a 2 × 2 contingency tables. Reviews, editorials, expert opinions, animal experiments, or studies presenting duplicate data were excluded.

The following information was retrieved from each study: first author, publication year, sample size, patient source (e.g., ED or ICU), time of age-adjusted qSOFA assessment, cutoff criteria of age-adjusted qSOFA, true positives, false positives, true negatives, and false negatives derived from the sensitivity and specificity of the age-adjusted qSOFA in predicting mortality and disease severity. When studies comprised multiple groups, each group was considered as an individual study.

### Quality assessment

Currently, there is no widely used assessment tool for assessing the quality of studies of predictive risk scores. This study used a revised seven-item quality assessment scale^27,28^, which was derived from the Quality Assessment of Diagnostic Accuracy Studies tool^29^ and Newcastle–Ottawa Scale^30^. It comprises seven criteria: unbiased patient selection; representative of a wide spectrum of disease severity; predictor variables assessed blinded to outcome; outcome assessed blinded to the predictor variables; accurate definition of outcomes; availability of the same clinical data; and adequate follow-up^27,28^. We defined adequate follow-up as a follow-up of > 90%. Two reviewers (SE and SHY) independently performed the methodological quality assessment. Any disagreements were resolved by discussion.

### Statistical analyses

Summary estimates of sensitivity, specificity, positive and negative likelihood ratios (LR+ and LR–), and pooled diagnostic odds ratio (DOR) were calculated using a bivariate random-effects model^31^. The DOR of a test (or score) is the ratio of the odds of positivity among patients versus the odds among healthy individuals or a control group^32,33^. When the DOR increases to greater than 1, the discriminative power of the outcome becomes greater^32^. We used summary receiver-operating characteristic (SROC) curves to calculate the area under the curve (AUC), which assisted in estimating the discriminative power of a test or score^33^. The AUC takes values between 0 and 1, with higher values indicating better test (or score) performance^34^. Heterogeneity of sensitivity and specificity were evaluated from the forest plots of the studies’ estimates and using a χ^2^ test (*P* < 0.1, significant). In the presence of significant heterogeneity, we conducted meta-regression analysis and a priori planned subgroup analysis to explore the sources of heterogeneity using the following as covariates with 95% confidence interval (CI): patient source (ED vs. ICU); sample size (< 10,000 vs. ≥ 10,000); outcome (mortality vs. disease severity); scales for assessing mental status in age-adjusted qSOFA (GCS vs. Alert, Voice, Pain, Unresponsive [AVPU] scale); age-specific vital signs criteria (2005 IPSCC definition vs. others); center (single center vs. multicenter); and cut-off value (≥ 2 vs. ≥ 1). We excluded studies in the meta-regression analysis if they used both the GCS and AVPU scale for mental status checks, or if their primary outcome was both in-hospital mortality and disease severity concomitantly. In addition, we also performed pooled analysis using one study population per study to examine whether the results were biased by including the same populations multiple times. As the reason for separation into different datasets from a study varies (e.g., outcomes, cutoff, age-specific vital signs criteria), we selected the data using the cutoff value of ≥ 2 and 2005 IPSCC definition as age-specific vital sign criteria. We measured publication bias with visualization of funnel plots and Egger’s test. Statistical analyses and meta-analyses were conducted using R program, version 3.6.3 (R Foundation for Statistical Computing, Vienna, Austria); *P-*values < 0.05 were considered statistically significant.

## Results

**Figure 1:**
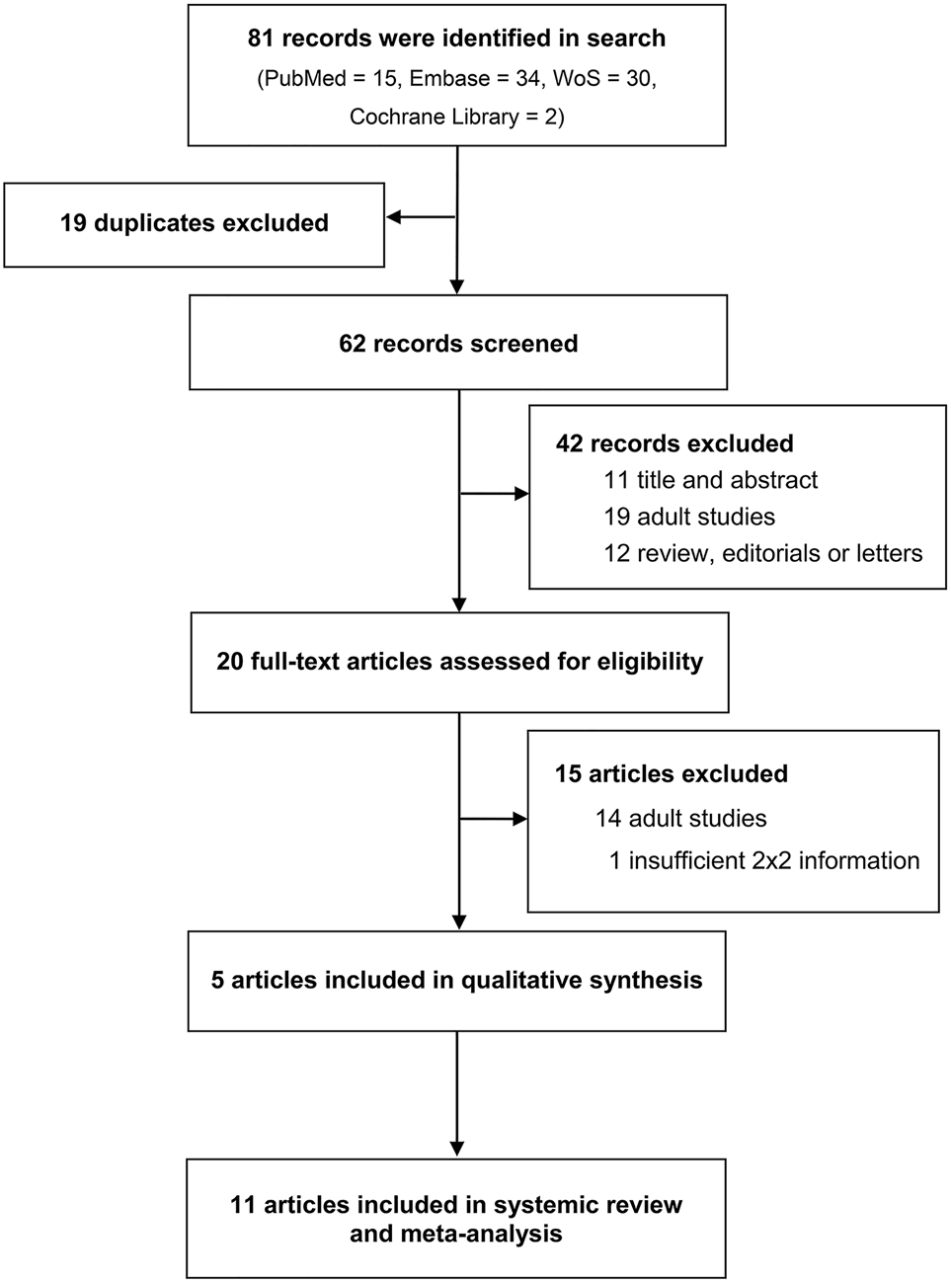
Flow diagram of the search and selection process.

PubMed, EMBASE, Web of Science and Cochrane database searches as per the predefined search words revealed 81 articles. After removing duplicates and screening abstracts, 20 full-text articles were read, resulting in 11 articles that met the inclusion criteria for the systematic review and meta-analysis. Reasons for exclusion are shown in the flow diagram (Fig. 1). Data from 172,569 patients of 11 observational studies^13,23–25,35^ were finally included. The general characteristics of the included studies are presented in Table 1 and Supplementary Table S1.

**Table 1:** Characteristics of the included studies.

Study	Country	Source	Age-specific vital signs criteria	Scales for mental status	Outcome	Center	Age-adjusted qSOFA cutoff	Enrolled patients (n)
2018 Peters—a^25^	USA and Canada	ICU	2005 IPSCC	GCS	In-hospital mortality	Multicenter	≥ 2	40,228
2018 Peters—b^25^	USA and Canada	ICU	PALS	GCS	In-hospital mortality	Multicenter	≥ 2	40,228
2018 Peters—c^25^	USA and Canada	ICU	PELOD2_MV	GCS	In-hospital mortality	Multicenter	≥ 2	40,228
2018 Schlapbach^13^	Australia and New Zealand	ICU	2005 IPSCC	GCS	In-hospital mortality	Multicenter	≥ 2	2259
2018 van Nassau^23^	Netherlands	ED	2005 IPSCC	AVPU or GCS	ICU transfer and/or mortality within 30 days	Single center	≥ 2	484
2018 Zallocco—a^35^	Italy	Pediatric tertiary referral center	2005 IPSCC	GCS	Development of severe sepsis^a^	Single center	≥ 2	89
2018 Zallocco—b^35^	Italy	Pediatric tertiary referral center	2005 IPSCC	GCS	ICU admission	Single center	≥ 2	89
2020 Romaine—a^24^	UK	ED	2005 IPSCC	AVPU	Critical care admission within 48 h	Single center	≥ 2	12,241
2020 Romaine—b^24^	UK	ED	2005 IPSCC	AVPU	Critical care admission within 48 h	Single center	≥ 1	12,241
2020 Romaine—c^24^	UK	ED	2005 IPSCC	AVPU	Sepsis-related mortality^b^	Single center	≥ 2	12,241
2020 Romaine—d^24^	UK	ED	2005 IPSCC	AVPU	Sepsis-related mortality^b^	Single center	≥ 1	12,241

AVPU, Alert, Voice, Pain, Unresponsive scale; ED, emergency department; GCS, Glasgow Coma Scale; ICU, intensive care unit; IPSCC, International Pediatric Sepsis Consensus Conference; PALS, Pediatric Advanced Life Support; PELOD2_MV, Pediatric Logistic Organ Dysfunction 2 with the use of mechanical ventilation; qSOFA, quick Sequential Organ Failure Assessment score; UK, United Kingdom; USA, United States of America.

^a^Defined by the 2005 International Pediatric Sepsis Consensus Conference definition.

^b^Defined as critical care admission within 48 h of ED visit with suspected sepsis, which led to in-hospital death within 28 days of admission.

### Study characteristics

The majority of studies were retrospective, and only one study^13^ was prospective. Six studies^13,24,25^ were designed to evaluate the value of the age-adjusted qSOFA in predicting mortality. Four studies^24,35^ evaluated the performance of the age-adjusted qSOFA in predicting disease severity; three studies^24,35^ evaluated the value of qSOFA in predicting ICU admission, and one study^35^ evaluated the value of qSOFA in predicting the development of severe sepsis^11^. Only one study^23^ was designed to evaluate the ability of the age-adjusted qSOFA to predict both ICU transfer and/or mortality within 30 days as the primary outcome.

Patient sources were as follows: 122,943 ICU patients from four studies^13,25^, 49,448 ED patients from five studies^23,24^, and 178 pediatric tertiary referral center patients from two studies^35^. The majority of the studies were single-center studies (n = 7, 63.6%)^23,24,35^ and chose cut-off criteria as ≥ 2 (n = 9, 81.8%)^13,23–25,35^. Most studies (n = 9, 81.8%)^13,23–25,35^ adopted the 2005 IPSCC definition for age-specific vital signs criteria. Six (54.5%) ^13,25,35^ studies used GCS, four studies (36.4%)^24^ used AVPU, and one study^23^ used either GCS or AVPU to assess mental status. All of the studies were published between 2018 and 2020 (Table 1 and Supplementary Table S1).

### Quality assessment of the included studies

Three studies (27.3%) enrolled patients consecutively^25^, and one single-center study^23^ defined suspected bacterial infection as the commencement of antibiotics within 24 h after ED arrival at the non-academic facility and excluded surgical diagnoses; thus, the study was deemed not representative of a wide spectrum of disease severity. Although all studies assessed the predictor variables that constituted the age-adjusted qSOFA blinded to outcomes, no studies clearly reported that the outcomes were assessed blindly to the age-adjusted qSOFA. Overall, outcomes were clearly defined and the same clinical data was available in all studies. All included studies showed adequate follow-up of patients (Supplementary Table S2).

### Age-adjusted qSOFA for predicting mortality and disease severity

**Figure 2:**
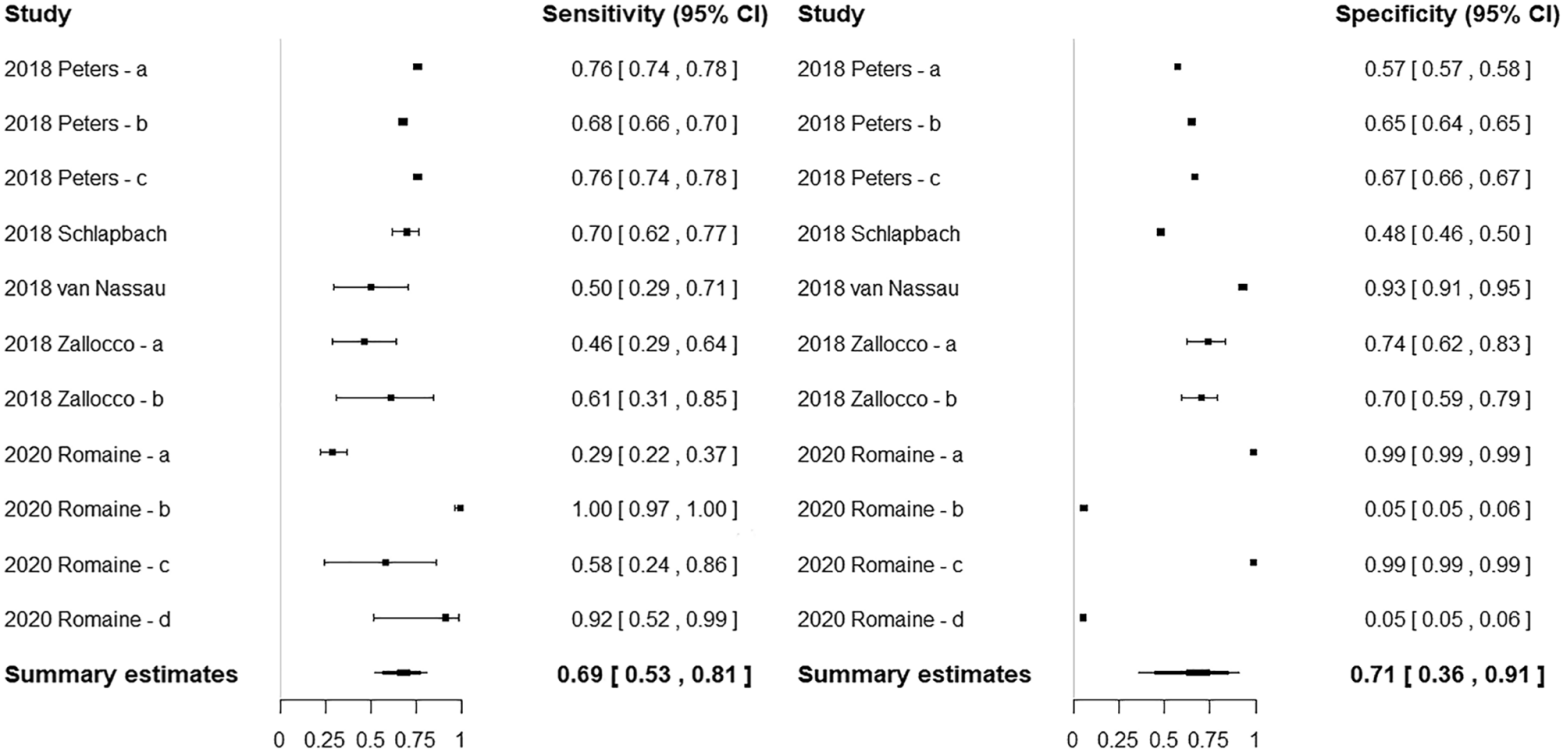
Coupled forest plots of the sensitivity and specificity of the age-adjusted quick Sequential Organ Failure Assessment score for predicting mortality and disease severity in pediatric patients with suspected or confirmed infection.

**Figure 3:**
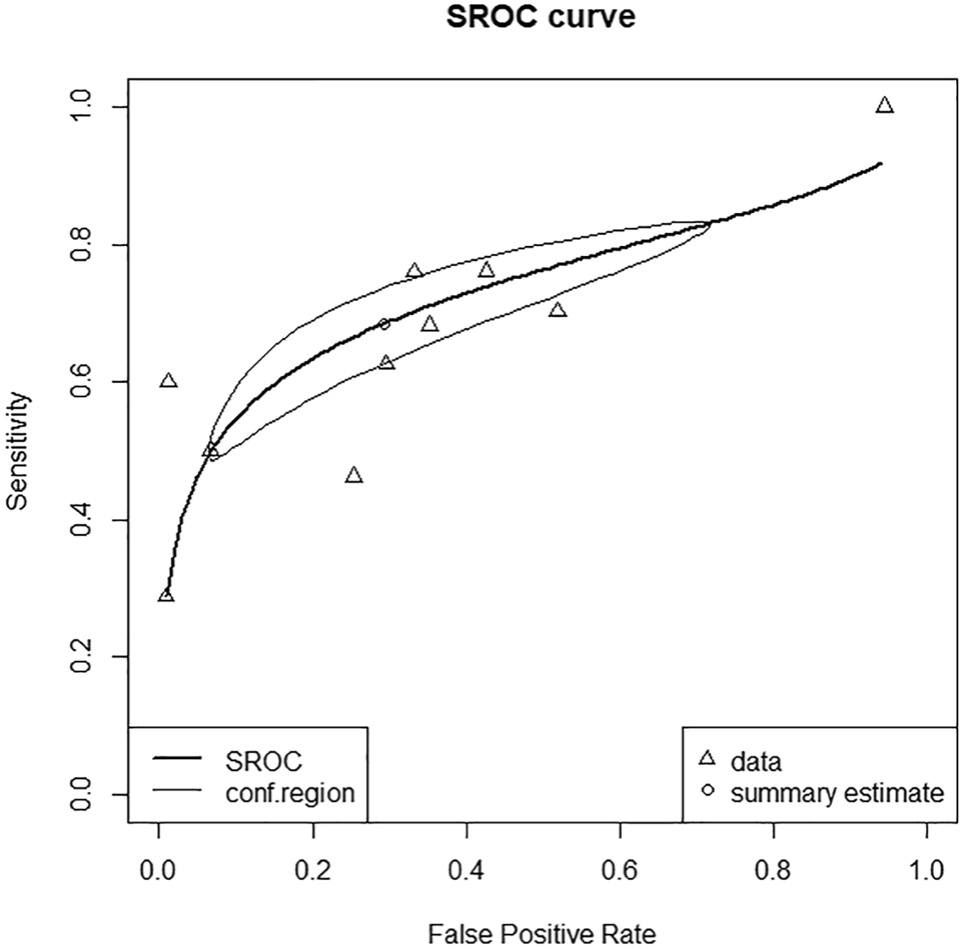
Summary receiver-operating characteristic (SROC) curve of the predictive performance of age-adjusted quick Sequential Organ Failure Assessment score for mortality and disease severity in pediatric patients with suspected or confirmed infection. The area under the curve of the SROC was 0.733 (95% CI 0.683–0.768). conf.region, 95% confidence region for SROC curve.

Included studies showed a wide range of sensitivities (0.29–1.00) and specificities (0.05–0.99) (Fig. 2 and Supplementary Table S3). Pooled sensitivity and specificity of qSOFA for predicting mortality and disease severity are 0.685 (95% CI 0.527–0.809) and 0.706 (95% CI 0.362–0.911), respectively. Pooled LR+, LR–, and DOR were 2.919 (95% CI 2.186–3.898), 0.519 (95% CI 0.428–0.630), and 6.565 (95% CI 4.459–9.667), respectively. The AUC was 0.733 (95% CI 0.683–0.768) (Fig. 3). Significant heterogeneity was noted in terms of sensitivity (χ^2^ = 233.079; *P* < 0.001) and specificity (χ^2^ = 47,040.96; *P* < 0.001). No significant publication bias was found in the funnel plot and Egger’s test (*P* = 0.8671) (Supplementary Fig. S1). When including one study population per study, five studies with a total of 55,301 patients were included and analyzed. Compared with the original results, the pooled sensitivity was slightly lower (0.685 vs. 0.571), and pooled specificity was higher (0.706 vs. 0.851). However, the pooled results of DOR (6.565 vs. 6.816) and AUC (0.733 vs. 0.711) were similar (Supplementary Tables S4 and S5 and Supplementary Figs. S2–S4).

### Heterogeneity exploration and subgroup analysis

A meta-regression analysis revealed that patient source, sample size, outcome, center, and scales for assessing mental status were significant factors affecting heterogeneity (Supplementary Table S6). When comparing summary estimates of the DOR between subgroups, significant differences were only found in relation to patient source and scales for assessing mental status (Table 2). Age-adjusted qSOFA showed a significantly higher pooled DOR in the studies including ED patients than in the studies including ICU patients [22.214 (95% CI 7.115–69.360) vs. 4.092 (95% CI 3.058–5.474), *P* = 0.005]; Studies that used the AVPU to assess mental status also showed a significantly higher pooled DOR compared with studies that used the GCS [23.009 (95% CI 4.559–116.123) vs. 3.968 (95% CI 3.015–5.224), *P* = 0.036].

**Table 2:** Subgroup analysis.

Covariates	Subgroup	Number of studies	DOR	95% CI	*P*-value
Patient source	ICU	4	4.092	3.058–5.474	**0.005**
	ED	5	22.214	7.115–69.360	
Sample size	≥ 10,000	7	8.628	5.422–13.730	0.123
	< 10,000	4	3.949	1.642–9.499	
Outcome	Mortality	6	4.372	3.192–5.989	0.448
	Severity	4	9.099	1.409–58.743	
Scales for assessing mental status	GCS	6	3.968	3.015–5.224	**0.036**
	AVPU	4	23.009	4.559–116.123	
Center	Multicenter	4	4.092	3.058–5.474	0.132
	Single center	7	10.925	3.145–7.958	
Age-specific vital signs criteria	2005 IPSCC definition	9	7.717	3.233–18.422	0.388
	Others	2	4.999	3.147–7.941	
Cut off value	≥ 2	9	6.676	4.508–9.888	0.694
	≥ 1	2	3.229	0.138–75.410	

Bold values denote statistical significance at the *P* < 0.05 level.

AVPU, Alert, Voice, Pain, Unresponsive scale; CI, confidence interval; DOR, diagnostic odds ratio; ED, Emergency Department; GCS, Glasgow Coma Scale; ICU, Intensive Care Unit; IPSCC, International Pediatric Sepsis Consensus Conference.

### Age-adjusted qSOFA for mortality

Six studies assessed the performance of age-adjusted qSOFA for predicting mortality. Pooled sensitivity and specificity of age-adjusted qSOFA for predicting mortality were 0.729 (95% CI 0.655–0.792) and 0.626 (95% CI 0.206–0.915), respectively. Pooled LR+, LR– and DOR were 1.925 (95% CI 1.629–2.275), 0.452 (95% CI 0.381–0.535), and 4.433 (95% CI 3.223–6.097), respectively. AUC was found to be 0.735 (95% CI 0.677–0.780) (Table 3). Further detailed accuracy estimates, coupled forest plots, SROC curves, and heterogeneity test results of studies evaluating predictive accuracy of the age-adjusted qSOFA for mortality are provided in the Supplementary Tables S7 and S8 and Supplementary Figs. S5–S7.

**Table 3:** Summary estimates of the predictive accuracy of the age-adjusted quick Sequential Organ Failure Assessment score according to the outcome.

Predictive performance	Mortality	Disease severity
Sensitivity (95% CI)	0.729 (0.655–0.792)	0.731 (0.207–0.966)
Specificity (95% CI)	0.626 (0.206–0.915)	0.724 (0.113–0.982)
Positive likelihood ratio (95% CI)	1.925 (1.629–2.275)	3.341 (0.498–22.416)
Negative likelihood ratio (95% CI)	0.452 (0.381–0.535)	0.708 (0.618–0.811)
Diagnostic odds ratio (95% CI)	4.433 (3.223–6.097)	8.866 (1.355–58.035)
AUC of SROC curve (95% CI)	0.735 (0.677–0.780)	0.786 (0.518–0.905)

AUC, area under the curve; CI, confidence interval; SROC, summary receiver-operating characteristic.

### Age-adjusted qSOFA for disease severity

Four studies reported the performance of age-adjusted qSOFA for predicting disease severity. Pooled sensitivity and specificity of age-adjusted qSOFA for predicting disease severity was 0.731 (95% CI 0.207–0.966) and 0.724 (95% CI 0.113–0.982). Pooled LR+, LR–, and DOR were 3.341 (95% CI 0.498–22.416), 0.708 (95% CI 0.618–0.811), and 8.866 (95% CI 1.355–58.035), respectively. The AUC was 0.786 (95% CI 0.518–0.905) (Table 3). Detailed accuracy estimates, coupled forest plots, SROC curves, and heterogeneity test results of studies evaluating the predictive accuracy of the age-adjusted qSOFA for disease severity are provided in the Supplementary Tables S9 and S10 and Supplementary Figs. S8–S10.

## Discussion

In this review, we assessed the performance of the age-adjusted qSOFA in predicting mortality and disease severity in pediatric patients with suspected or confirmed infection. We identified 11 studies, including 172,569 patients from the ED, pediatric tertiary referral center, and ICU. We found that the age-adjusted qSOFA had a moderate performance for predicting in-hospital mortality and disease severity in pediatric patients.

The qSOFA was initially recommended by the SEPSIS-3 task force as a readily available bedside tool^16,36^, and the age-adjusted qSOFA has the same advantages: it does not require laboratory tests and enables prompt and repeatable assessment of patients. However, as a screening tool to identify ‘at-risk patients’, the age-adjusted qSOFA satisfies the requirements for convenience and feasibility, but does not satisfy the requirement for high sensitivity^37^. In clinical practice, screening tools typically require high sensitivity to safely rule out those at low risk of adverse outcomes^38^.

Determining which patients are at high risk of severe illness or mortality is essential for appropriate clinical decision making. When clinicians initially encounter pediatric patients with suspected infection, the specific outcomes (e.g. mortality, ICU admission or prolonged hospital admission itself) would be not matter at that moment, only whether this patient has a potential to become a severe, critical patient requiring close observation, and intensive treatment will be of more interest to clinicians. Thus, we intended to assess the predictive performance of age-adjusted qSOFA as a quick, easy, bedside screening tool for identifying these ‘at risk patients’. Then, we demonstrated the individual performance of age-adjusted qSOFA according to the specific outcomes, such as mortality and disease severity, for clinicians to consider further prognostic aspects.

As described in previous studies^39–41^, we assessed the discriminative power of the prediction score (age-adjusted qSOFA) for identifying at-risk pediatric patients by calculating AUC. An AUC above 0.7 was considered to be acceptable and useful^34,40^. In our results, aged-adjusted qSOFA achieved an AUC of 0.733, indicating a useful discrimination for pediatric patients at risk who need close monitoring and intensive treatment.

Likewise, the DOR was also calculated as another single indicator of age-adjusted qSOFA performance for discrimination of at-risk patients^42^. DOR of 6.57 in our result means that the odds of positivity (above cutoff value of age-adjusted qSOFA) in at risk patients is about six times higher than the odds of positivity in non-risk patients. DOR does not depend on disease prevalence^33^. However, it depends on what criteria are used to define disease or pathological conditions of the study population (e.g., comorbidity, disease severity)^33^. Because considerable heterogeneity existed in our analysis, we conducted the subgroup analysis of DOR of age-adjusted qSOFA according to the various factors that can affect the results and also the causes of the heterogeneity in the pooled analysis.

Regarding patient sources, qSOFA has reported a better predictive power to that of the full SOFA for in-hospital mortality in adult patients outside the ICU^16,17^. However, the full SOFA showed higher predictive validity when compared with the qSOFA among patients in the ICU^16,17^. The majority of patients in ICU are administered vasopressor support and/or mechanical ventilation, thus the qSOFA may not have a reasonable clinical value for patients in this setting^17^. Our results also found that the age-adjusted qSOFA has a better DOR for predicting mortality and disease severity in ED patients than in ICU patients. These results showed that the age-adjusted qSOFA is more useful for screening pediatric patients outside the ICU.

Scales assessing mental status is a significant source of heterogeneity in this meta-analysis. Currently, the qSOFA in adults uses the GCS^16^. In our analysis, studies using the AVPU to assess mental status showed higher predictive performance than studies using the GCS. The AVPU scale is less complex than the GCS, and uses only four categories (Alert; Verbal response; response to Pain; Unresponsive). The AVPU scale can be used quickly and easily^43^ and has been reported to correlate well with the GCS^44^. According to the results of this study, it is reasonable to use the AVPU scale to assess mental status in the age-adjusted qSOFA.

However, there are important limitations to the application of the age-adjusted qSOFA in the pediatric field. First, there is a global tendency not to measure blood pressure in pediatric acute care settings^45^. In addition, hypotension presents at a late stage of septic shock in pediatric patients^7,46^. Unlike adults, blood pressure is typically maintained in children in the early stage of septic shock, compensated by increased heart rate and systemic vascular resistance^47–49^. Thus, it may not be a valuable measure in frontline health care facilities such as ED^47^.

To address these limitations, Romaine et al.^24^ suggested a novel scale, the “Liverpool quick Sequential Organ Failure Assessment (LqSOFA)” score. The LqSOFA score, which ranges 0–4, comprises respiratory rate, age-adjusted heart rate, capillary refill time, and level of consciousness assessed using the AVPU scale. Romaine et al.^24^ reported that LqSOFA with ≥ 2 criteria showed equal sensitivity (0.6) and specificity (0.988) for predicting sepsis-related mortality when compared with an age-adjusted qSOFA with ≥ 2 criteria. In addition, when compared with age-adjusted qSOFA ≥ 2 criteria, LqSOFA ≥ 2 criteria showed low but better sensitivity (0.392 vs. 0.289) and similar high specificity (0.992 vs. 0.991) for the prediction of critical care admission within 48 h.

When comparing SIRS to qSOFA criteria, recent meta-analyses have consistently presented a higher sensitivity but lower specificity of SIRS criteria than those of the qSOFA for the prediction of in-hospital mortality among adult patients in various clinical settings^39,50–53^. In this review, we could not compare the pooled predictive performance of the age-adjusted qSOFA with that of the SIRS criteria, because there were few pediatric studies that provided the required data. Schlapbach et al.^13^ compared the predictive performance of the age-adjusted qSOFA with that of SIRS criteria for in-hospital mortality and showed that the age-adjusted qSOFA had a lower sensitivity but higher specificity and LR+ than those of SIRS criteria. Higher specificity and LR+ indicate that the age-adjusted qSOFA is a better scale for ruling in pediatric patients at risk of mortality. Regarding disease severity, van Nassau et al.^23^ reported that age-adjusted qSOFA ≥ 2 criteria showed lower sensitivity and LR+ but higher specificity than those of SIRS ≥ 2 criteria in predicting prolonged hospitalization (length of stay ≥ 7 days).

The present study has several strengths. As far as we are aware, our review is the first systematic review and meta-analysis to evaluate the predictive performance of the age-adjusted qSOFA in pediatric patients. Our meta-analyses used data from favorable quality studies with a large sample size. This may provide the substrate for future guidelines for screening infectious pediatric patients who are likely to progress to severe disease, or who are at risk of death.

This study has some limitations. First, there is a significant heterogeneity in this meta-analysis. We investigated the factors affecting heterogeneity by meta-regression and subgroup analysis, although we could not investigate factors related to differing diagnostic criteria and specific clinical settings. Second, if the predictive ability of the age-adjusted qSOFA was assessed with different outcomes or with different criteria in a same cohort, we consider them as separate studies^54^. Because the pooled results of AUC and DOR were similar between results using one study population per study and results using several datasets from the same study population, the overall predictive power of the age-adjusted qSOFA for mortality and morbidity can be considered similar in both analyses. This also indicates that the results would not to be strongly biased by including the same populations multiple times. Nevertheless, our results still need to be interpreted and applied cautiously because the same study population were pooled. Third, most of the included studies are retrospective studies, which were not devised to the validate the age-adjusted qSOFA. Fourth, most of the studies were conducted in western countries. Further studies are required to ensure the applicability of the results of studies of the age-adjusted qSOFA to other countries. Fifth, we did not search gray literature, as we aimed to review the characteristics of published literature. Incorporating a gray literature search may help to minimize the effects of publication bias^55^; however, we found no significant publication bias in this analysis. Sixth, long-term outcomes or healthcare costs were not available in the literature that was included; thus we could not evaluate these in this analysis. Finally, we could not compare the overall predictive performance of the age-adjusted qSOFA with other predictive biomarkers, due to the limited number of clinical studies.

## Conclusions

Current evidence suggests that the age-adjusted qSOFA has a moderate predictive value for mortality and disease severity in pediatric patients with suspected or confirmed infection. The age-adjusted qSOFA is a simple and feasible tool to use in settings outside the ICU such as ED, and in resource-limited settings. However, a screening tool with higher sensitivity for pediatric patients is needed.

## Supplementary Information

Supplementary Information.

## Data Availability

All data generated or analyzed during this systematic review are included in this manuscript (and its Supplementary Materials).
