# Supplementary material for: Age-adjusted quick Sequential Organ Failure Assessment score for predicting mortality and disease severity in children with infection: a systematic review and meta-analysis
Source: Sci Rep. 2021 Nov 4;11:21699. doi: 10.1038/s41598-021-01271-w (PMC8568945; doi:10.1038/s41598-021-01271-w)
Supplement: Supplementary file 1 — Supplementary Information. [file 41598_2021_1271_MOESM1_ESM.pdf]

Supplementary Materials

Age-adjusted quick Sequential Organ Failure Assessment score for predicting mortality and disease severity in children with infection: a systematic review and meta-analysis

Sohyun Eun[1], Haemin Kim[1], Ha Yan Kim[2], Myeongjee Lee[2], Go Eun Bae[3], Heoungjin Kim[1], Chung Mo Koo[1], Moon Kyu Kim[1] and Seo Hee Yoon[1,*]

[1] Department of Pediatrics, Severance Children's Hospital, Yonsei University College of Medicine, Seoul, Korea

[2] Biostatistics Collaboration Unit, Department of Biomedical Systems Informatics, Yonsei University College of Medicine, Seoul, Korea

[3] Department of Emergency Medicine, Yonsei University College of Medicine, Seoul, Korea

* Correspondence: Seo Hee Yoon, E-mail: yoonsh@yuhs.ac; Tel.: +82-2-2227-7963.

Table S1: Characteristics of included studies.

Study	Type of study	Study period	Age of inclusion	Time of age-adjusted qSOFA assessment	Inclusion criteria	Exclusion criteria	Definition of suspected or confirmed cases of infection	Reason for separation into different dataset studies
**2018 Peters - a**	Retrospective	2009–2014	<18 years	Within 12 hours of admission at the PICU	All pediatric patients (age < 18 years), admitted to the 130 PICUs in North America and who accepted to participate in the study, and who were assigned a diagnostic code indicative of infection upon discharge	All patients with missing data	STAR†/ICD-9-codes indicative of an infection on discharge (http://links.lww.com/PCC/A670)	Age-specific vital signs criteria: 2005 IPSCC
**2018 Peters - b**	Retrospective	2009–2014	<18 years	Within 12 hours of at the PICU admission	All pediatric patients (age < 18 years), admitted to the 130 PICUs in North America who accepted to participate in the study and were assigned a diagnostic code indicative of an infection on discharge	All patients with missing data	STAR†/ICD-9-codes indicative of an infection on discharge (http://links.lww.com/PCC/A670)	Age-specific vital signs criteria: PALS
**2018 Peters - c**	Retrospective	2009–2014	<18 years	Within 12 hours of PICU admission	All pediatric patients (age < 18 years),	All patients with missing data	STAR†/ICD-9-codes	Age-specific vital signs

					admitted to the 130 PICUs in North America, who were assigned a diagnostic code indicative of an infection on discharge		indicative of an infection on discharge (http://links.lww.com/PCC/A670)	criteria: PELOD2_MV
**2018 Schlapbach**	Prospective	2000–2016	<18 years	During the first 24 hours of ICU admission	Patients < 18 years admitted to the ICUs in Australia and New Zealand who presented with suspected or proven infection at admission to an adult or combined adult/pediatric ICU which contributed data to the ANZICS Adult Patient Database	Duplicated record, patients > 16 years who were transferred alive to another ICU with an unknown outcome, and missing outcome data	Infection-related diagnoses according to the ANZICS modification of the Acute Physiology and Chronic Health Evaluation (APACHE) III at admission to ICU; sepsis was defined according to the American College of Chest Physicians–Society of Critical Care Medicine consensus definition (1992)	N/A
**2018 van Nassau**	Retrospective	March 2013 to January 2018	<18 years	First values measured within 24 hours after entry at the ED	Patients <18 years who visited the ED and were subsequently	Patients admitted with a surgical diagnosis were excluded	Suspected bacterial infection was defined as the initiation of therapeutic	N/A

					admitted to the pediatric ward with a suspected bacterial infection		antibiotic therapy* within 24 hours after entry at the ED	
**2018 Zallocco - a**	Retrospective	January 1, 2006, to December 31, 2016	Median, 12 months (IQR, 1–75 months)	During the first 24 hours after the onset of sepsis; the onset time for sepsis was defined as the earliest time during a hospital encounter when the patient met the criteria for sepsis	Pediatric patients with sepsis admitted to a pediatric tertiary referral center	Early-onset sepsis (neonates less than 72 hours old), and patients who do not fulfill the criteria for sepsis	Sepsis was defined according to the 2005 IPSCC definition; ICD-9-CM codes for sepsis (995.91); neonatal sepsis (771.81); severe sepsis (995.92), septic shock (785.52), and septicemia (038.9) at discharge were used to generate the lists of potential cases	Outcome: development of severe sepsis
**2018 Zallocco - b**	Retrospective	January 1, 2006, to December 31, 2016	Median, 12 months (IQR, 1–75 months)	During the first 24 hours after the onset of sepsis; the onset time for sepsis was defined as the earliest time during a hospital encounter when the patient met the criteria for sepsis	Pediatric patients with sepsis admitted to a pediatric tertiary referral center	Early-onset sepsis (neonates less than 72 hours old), and patients who do not fulfill the criteria for sepsis	Sepsis was defined according to the 2005 IPSCC definition; ICD-9-CM codes for sepsis (995.91); neonatal sepsis (771.81); severe sepsis (995.92), septic shock	Outcome: ICU admission

							(785.52), and septicemia (038.9) at discharge were used to generate the lists of potential cases	
**2020 Romaine - a**	Retrospective	September 1, 2015, to August 31, 2017	<16 years	The worst observations recorded in the ED	Febrile children who presented to the ED	Missing observation data ≥2 components, no history of fever, and transfer from another hospital	Febrile children (fever was defined as a temperature ≥38°C recorded in the ED or a history of fever reported in the previous 3 days)	Outcome: critical care admission within 48 h;<br><br>Age-adjusted qSOFA cutoff: ≥ 2
**2020 Romaine - b**	Retrospective	September 1, 2015, to August 31, 2017	<16 years	The worst observations recorded in the ED	Febrile children who presented to the ED	Missing observation data ≥2 components, no history of fever, and transfer from another hospital	Febrile children (fever was defined as a temperature ≥38°C recorded in the ED or a history of fever reported in the previous 3 days)	Outcome: critical care admission within 48 h;<br><br>Age-adjusted qSOFA cutoff: ≥ 1
**2020 Romaine - c**	Retrospective	September 1, 2015, to August 31, 2017	<16 years	The worst observations recorded in the ED	Febrile children who presented to the ED	Missing observation data ≥2 components, no history of fever, and transfer from another hospital	Febrile children (fever was defined as a temperature ≥38°C recorded in the ED or a history of fever reported in the	Outcome: sepsis-related mortality;<br><br>Age-adjusted qSOFA cutoff: ≥ 2

							previous 3 days)	
**2020 Romaine - d**	Retrospective	September 1, 2015, to August 31, 2017	<16 years	The worst observations recorded in the ED	Febrile children who presented to the ED	Missing observation data ≥2 components, no history of fever, and transfer from another hospital	Febrile children (fever was defined as a temperature ≥38°C recorded at the ED or a history of fever reported in the previous 3 days)	Outcome: sepsis-related mortality;<br><br>Age-adjusted qSOFA cutoff: ≥ 1

ICD, International Classification of Diseases; CM, Clinical Modification; PICU, pediatric intensive care unit; ICU, intensive care unit; ED, emergency department; ANZICS, Australian and New Zealand Intensive Care Society; IQR, interquartile range; qSOFA, quick Sequential Organ Failure Assessment score; IPSCC, International Pediatric Sepsis Consensus Conference; PALS, Pediatric Advanced Life Support; PELOD2_MV, Pediatric Logistic Organ Dysfunction 2 with the use of mechanical ventilation; N/A, not applicable.

† STAR code is the proprietary diagnosis classification of the Virtual Pediatric Systems database.
*The authors considered 11 antibiotics as therapeutic: amoxicillin, amoxicillin-clavulanic acid, benzylpenicillin, cefotaxime, ceftazidime, ceftriaxone, cefuroxime, clarithromycin, clindamycin, flucloxacillin, and vancomycin.

Table S2: Quality assessment.

Author (year)	Selection of patients in an unbiased manner*	Representative of a wide spectrum of the severity of disease†	Predictor variables assessed blindly to outcome	Outcome assessed blindly to predictor variables‡	Accurate definition of outcomes	Availability of the same clinical data§	Adequate follow-up¶
**2018 Peters - a**	Yes	Yes	Yes	Not clear	Yes	Yes	Yes
**2018 Peters - b**	Yes	Yes	Yes	Not clear	Yes	Yes	Yes
**2018 Peters - c**	Yes	Yes	Yes	Not clear	Yes	Yes	Yes
**2018 Schlapbach**	Not clear	Yes	Yes	Not clear	Yes	Yes	Yes
**2018 van Nassau**	No	No	Yes	Not clear	Yes	Yes	Yes
**2018 Zallocco - a**	No	Yes	Yes	Not clear	Yes	Yes	Yes
**2018 Zallocco - b**	No	Yes	Yes	Not clear	Yes	Yes	Yes
**2020 Romaine - a**	No	Yes	Yes	Not clear	Yes	Yes	Yes
**2020 Romaine - b**	No	Yes	Yes	Not clear	Yes	Yes	Yes
**2020 Romaine - c**	No	Yes	Yes	Not clear	Yes	Yes	Yes
**2020 Romaine - d**	No	Yes	Yes	Not clear	Yes	Yes	Yes

*Consecutive or random samples.
† Representative of all patients at that site under a given condition.
‡ Without knowledge of the predictor variables comprising the age-adjusted quick sequential organ failure assessment score.
§ Available clinical data when interpreting age-adjusted quick sequential organ failure assessment scores were the same as those available in practice.
¶ We arbitrarily defined adequate follow-up as a follow-up rate of > 90%.

Table S3: Summary estimates of the overall predictive accuracy of age-adjusted qSOFA for mortality and disease severity.

Study	Sensitivity (95% CI)	Specificity (95% CI)	Positive LR (95% CI)	Negative LR (95% CI)	DOR (95% CI)
2018 Peters - a	0.761(0.740-0.780)	0.574(0.569-0.579)	1.785(1.733-1.837)	0.417(0.383-0.454)	4.279(3.821-4.792)
2018 Peters - b	0.682(0.659-0.704)	0.648(0.643-0.653)	1.938(1.871-2.007)	0.491(0.458-0.526)	3.947(3.557-4.381)
2018 Peters - c	0.759(0.738-0.779)	0.668(0.663-0.672)	2.284(2.216-2.355)	0.361(0.332-0.393)	6.328(5.651-7.087)
2018 Schlapbach	0.701(0.621-0.771)	0.480(0.459-0.501)	1.349(1.201-1.515)	0.622(0.48-0.806)	2.168(1.493-3.149)
2018 van Nassau	0.5(0.295-0.705)	0.933(0.906-0.952)	7.413(4.226-13.004)	0.536(0.342-0.841)	13.825(5.245-36.444)
2018 Zallocco - a	0.463(0.292-0.644)	0.742(0.623-0.833)	1.796(1.004-3.211)	0.724(0.495-1.057)	2.482(0.968-6.361)
2018 Zallocco - b	0.611(0.309-0.847)	0.701(0.595-0.789)	2.045(1.103-3.793)	0.555(0.242-1.273)	3.688(0.89-15.275)
2020 Romaine - a	0.29(0.221-0.372)	0.991(0.989-0.992)	31.822(23.07-43.895)	0.716(0.643-0.797)	44.439(29.348-67.29)
2020 Romaine - b	0.996(0.966-1.00)	0.055(0.051-0.059)	1.054(1.042-1.066)	0.067(0.004-1.07)	15.688(0.975-252.37)
2020 Romaine - c	0.583(0.241-0.861)	0.988(0.986-0.990)	48.725(24.314-97.645)	0.422(0.164-1.087)	115.541(22.612-590.389)
2020 Romaine - d	0.917(0.517-0.991)	0.054(0.050-0.058)	0.969(0.761-1.234)	1.539(0.108-21.892)	0.63(0.035-11.398)
**Bivariate summary estimates**	**0.685(0.527-0.809)**	**0.706(0.362-0.911)**	**2.919(2.186-3.898)**	**0.519(0.428-0.630)**	**6.565(4.459-9.667)**

CI, confidence interval; DOR, diagnostic odds ratio; LR, likelihood ratio; qSOFA, quick sequential organ failure assessment score.

Table S4: Summary estimates of the overall predictive accuracy of age-adjusted qSOFA for mortality and disease severity (one study population per each study).

Study	Sensitivity (95% CI)	Specificity (95% CI)	Positive LR (95% CI)	Negative LR (95% CI)	DOR (95% CI)
2018 Peters	0.761(0.740–0.781)	0.574(0.569–0.579)	1.785(1.734–1.838)	0.417(0.383–0.454)	4.283(3.824–4.797)
2018 Schlapbach	0.703(0.622–0.773)	0.480(0.459–0.501)	1.352(1.204–1.518)	0.619(0.477–0.803)	2.184(1.501–3.177)
2018 van Nassau	0.500(0.290–0.710)	0.933(0.907–0.953)	7.516(4.235–13.339)	0.536(0.337–0.851)	14.032(5.198–37.884)
2018 Zallocco	0.462(0.288–0.645)	0.746(0.627–0.837)	1.817(1.005–3.288)	0.722(0.492–1.060)	2.518(0.967–6.558)
2020 Romaine	0.289(0.219–0.370)	0.991(0.989–0.992)	31.794(23.006–43.937)	0.718(0.644–0.799)	44.303(29.202–67.215)
**Bivariate summary estimates**	**0.571(0.383–0.741)**	**0.851(0.507–0.97)**	**3.964(2.042–7.694)**	**0.587(0.427–0.805)**	**6.816(2.415–19.237)**

CI, confidence interval; DOR, diagnostic odds ratio; LR, likelihood ratio; qSOFA, quick sequential organ failure assessment score.

Table S5: Heterogeneity among studies evaluating predictive accuracy of age-adjusted qSOFA for mortality and disease severity (one study population per study).

	Chi-squared	*p*-value
**Sensitivity**	151.378	< 0.001
**Specificity**	7725.837	< 0.001

qSOFA, quick sequential organ failure assessment score.

Table S6: Meta-regression analyses.

Covariates	DOR		
	**Estimate**	**Std. Error**	***p*-value**
Patient source	2.012	0.329	**<0.001**
Sample size	0.843	0.414	**0.042**
Outcome	-1.145	0.373	**0.002**
Scales for assessing mental status	-2.287	0.356	**<0.001**
Center	-1.319	0.327	**<0.001**
Age-specific vital signs criteria	0.405	0.567	0.450
Cut off value	0.694	1.101	0.528

DOR, diagnostic odds ratio; Std.Error, standard error.

Table S7: Summary estimates of the predictive accuracy of age-adjusted qSOFA for mortality.

Study	Sensitivity (95% CI)	Specificity (95% CI)	Positive LR (95% CI)	Negative LR (95% CI)	DOR (95% CI)
2018 Peters - a	0.761(0.740-0.780)	0.574(0.569-0.579)	1.785(1.733-1.837)	0.417(0.383-0.454)	4.279(3.821-4.792)
2018 Peters - b	0.682(0.659-0.704)	0.648(0.643-0.653)	1.938(1.871-2.007)	0.491(0.458-0.526)	3.947(3.557-4.381)
2018 Peters - c	0.759(0.738-0.779)	0.668(0.663-0.672)	2.284(2.216-2.355)	0.361(0.332-0.393)	6.328(5.651-7.087)
2018 Schlapbach	0.701(0.621-0.771)	0.480(0.459-0.501)	1.349(1.201-1.515)	0.622(0.48-0.806)	2.168(1.493-3.149)
2020 Romaine - c	0.583(0.241-0.861)	0.988(0.986-0.990)	48.725(24.314-97.645)	0.422(0.164-1.087)	115.541(22.612-590.389)
2020 Romaine - d	0.917(0.517-0.991)	0.054(0.050-0.058)	0.969(0.761-1.234)	1.539(0.108-21.892)	0.63(0.035-11.398)
**Bivariate summary estimates**	**0.729(0.655-0.792)**	**0.626(0.206-0.915)**	**1.925(1.629-2.275)**	**0.452(0.381-0.535)**	**4.433(3.223-6.097)**

DOR, diagnostic odds ratio; LR, likelihood ratio; qSOFA, quick sequential organ failure assessment score.

Table S8: Heterogeneity among studies evaluating predictive accuracy of age-adjusted qSOFA for mortality.

	Chi-squared	*p*-value
**Sensitivity**	37.664	< 0.001
**Specificity**	24357.25	< 0.001

qSOFA, quick sequential organ failure assessment score.

Table S9: Summary estimates of the predictive accuracy of age-adjusted qSOFA for disease severity.

Study	Sensitivity (95% CI)	Specificity (95% CI)	Positive LR (95% CI)	Negative LR (95% CI)	DOR (95% CI)
2018 Zallocco - a	0.463(0.292-0.644)	0.742(0.623-0.833)	1.796(1.004-3.211)	0.724(0.495-1.057)	2.482(0.968-6.361)
2018 Zallocco - b	0.611(0.309-0.847)	0.701(0.595-0.789)	2.045(1.103-3.793)	0.555(0.242-1.273)	3.688(0.89-15.275)
2020 Romaine - a	0.29(0.221-0.372)	0.991(0.989-0.992)	31.822(23.07-43.895)	0.716(0.643-0.797)	44.439(29.348-67.29)
2020 Romaine - b	0.996(0.966-1.00)	0.055(0.051-0.059)	1.054(1.042-1.066)	0.067(0.004-1.07)	15.688(0.975-252.37)
**Bivariate Summary estimates**	**0.731(0.207-0.966)**	**0.724(0.113-0.982)**	**3.341(0.498-22.416)**	**0.708(0.618-0.811)**	**8.866(1.355-58.035)**

CI, confidence interval; DOR, diagnostic odds ratio; LR, likelihood ratio; qSOFA, quick sequential organ failure assessment score.

Table S10: Heterogeneity among studies evaluating predictive accuracy of age-adjusted qSOFA for disease severity.

	Chi-squared	*p*-value
**Sensitivity**	148.244	< 0.001
**Specificity**	21292.29	< 0.001

qSOFA, quick sequential organ failure assessment score.

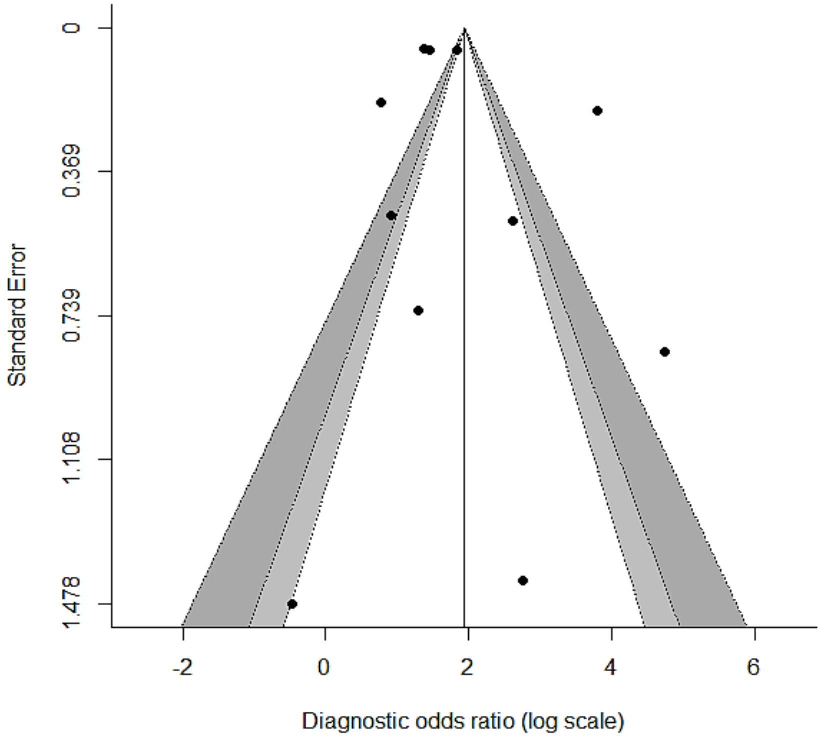

Figure S1: Funnel plot of the studies included in the current meta-analysis. No significant publication bias was detected by Egger's test (p=0.8671).

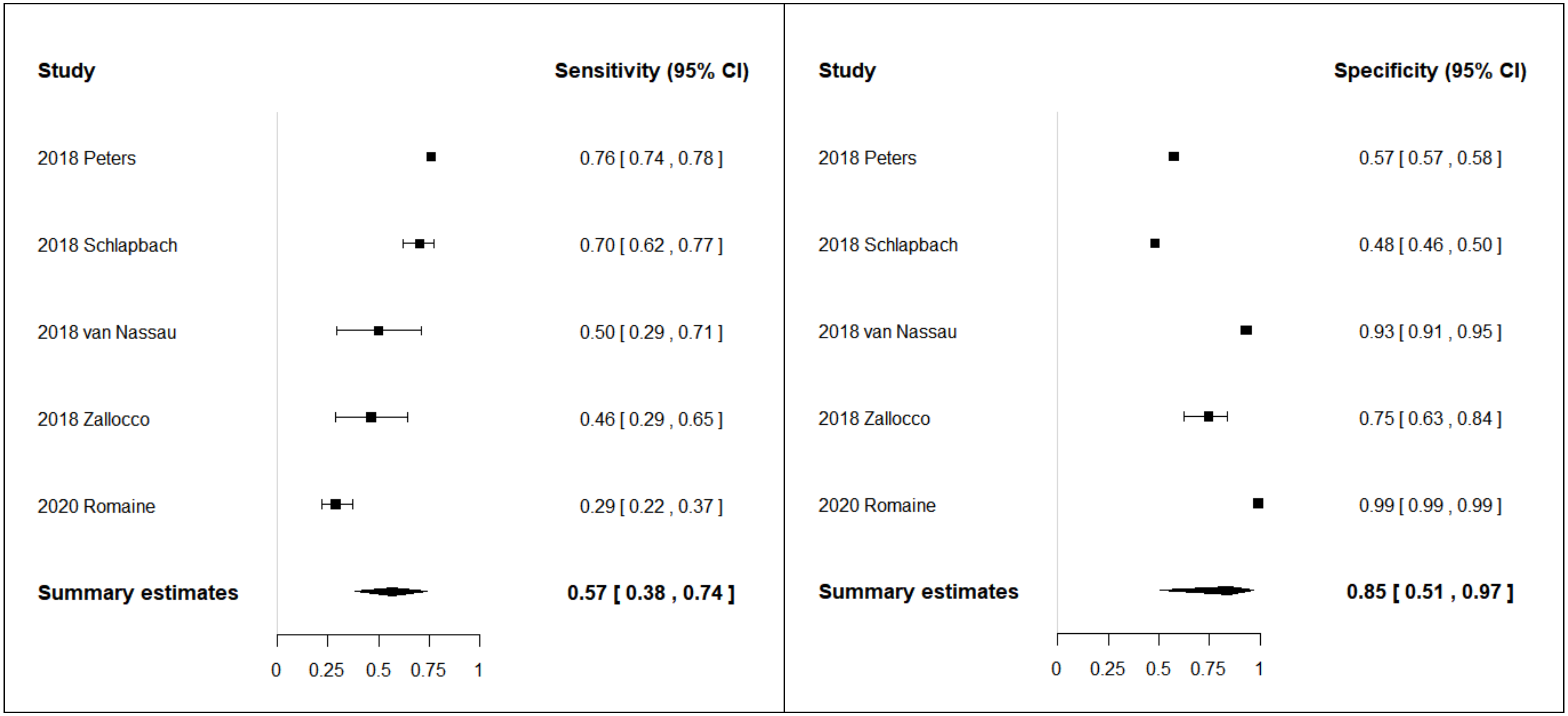

Figure S2: Coupled forest plots for sensitivity and specificity on predicting mortality and disease severity (one study population per study).

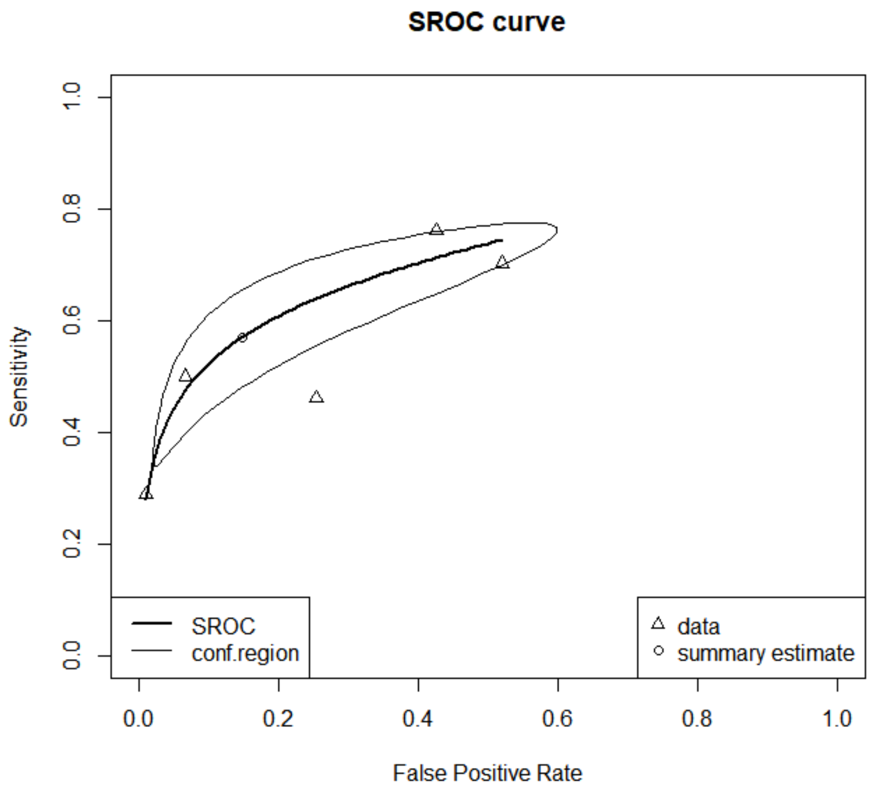

Figure S3: Summary receiver operating characteristic (SROC) curve of the predictive performance of age-adjusted quick sequential organ failure assessment score for mortality and disease severity (one study population per study). The area under the curve of the SROC was 0.711 (95% CI: 0.608–0.801). conf. region, 95% confidence region for SROC curve.

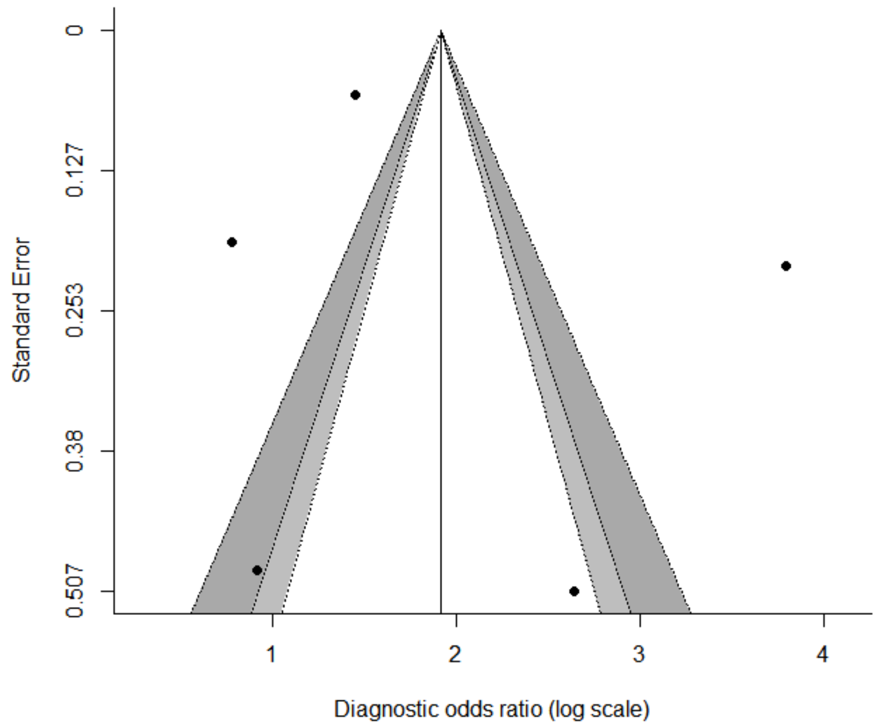

Figure S4: Funnel plot of the included studies (one study population per study). No significant publication bias was detected by Egger's test (p=0.9447).

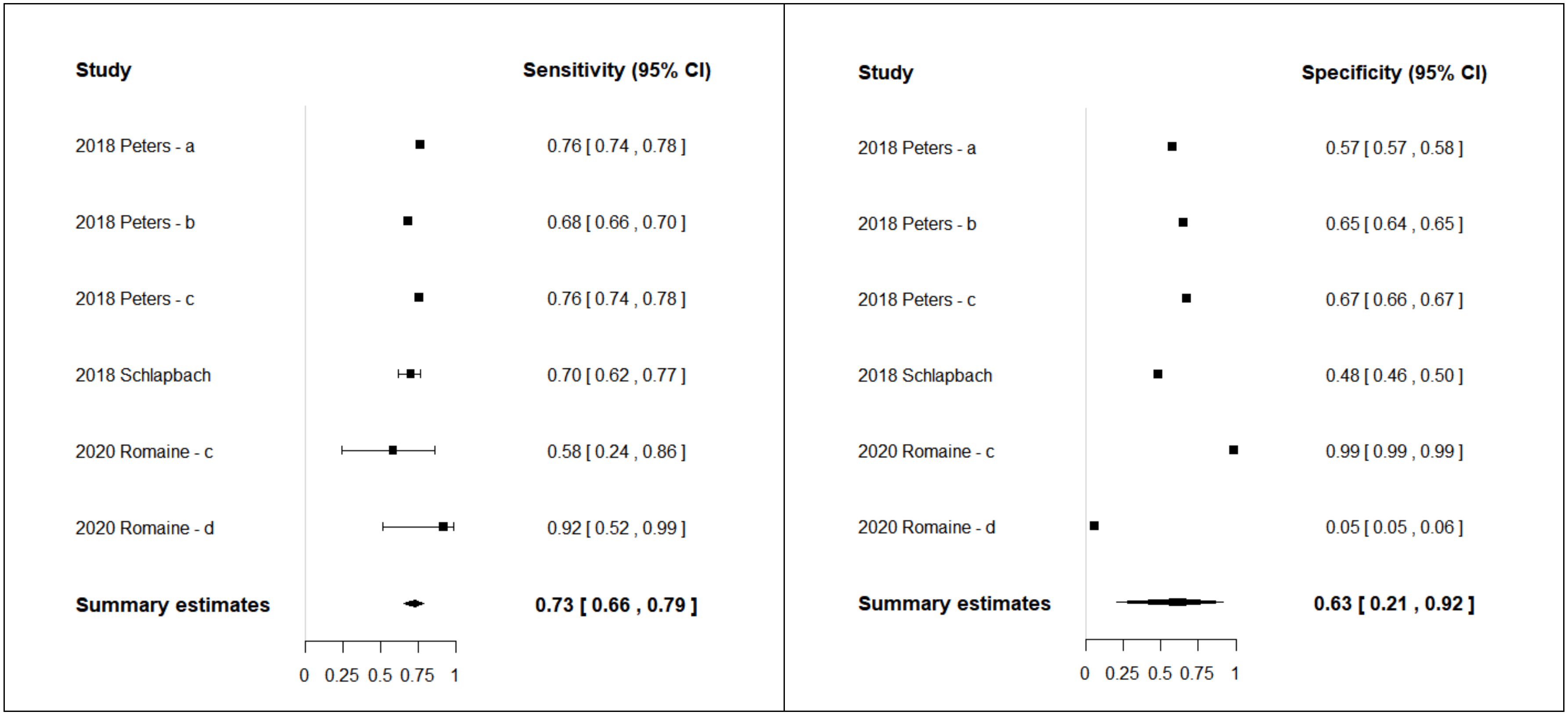

Figure S5: Coupled forest plots for sensitivity and specificity on predicting mortality.

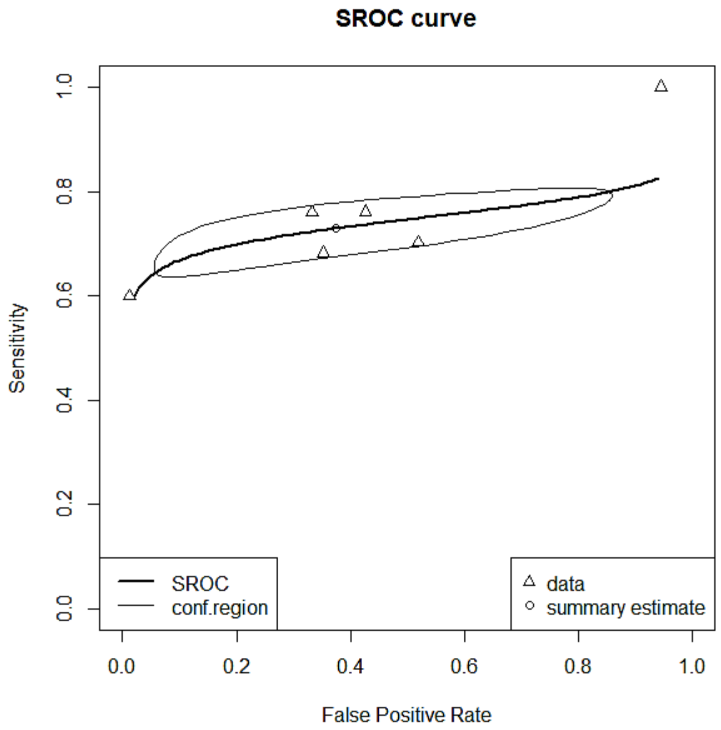

Figure S6: Summary receiver operating characteristic (SROC) curve of the predictive performance of age-adjusted quick sequential organ failure assessment score for mortality. The area under the curve of the SROC was 0.735 (95% CI: 0.677–0.780). conf. region, 95% confidence region for SROC curve.

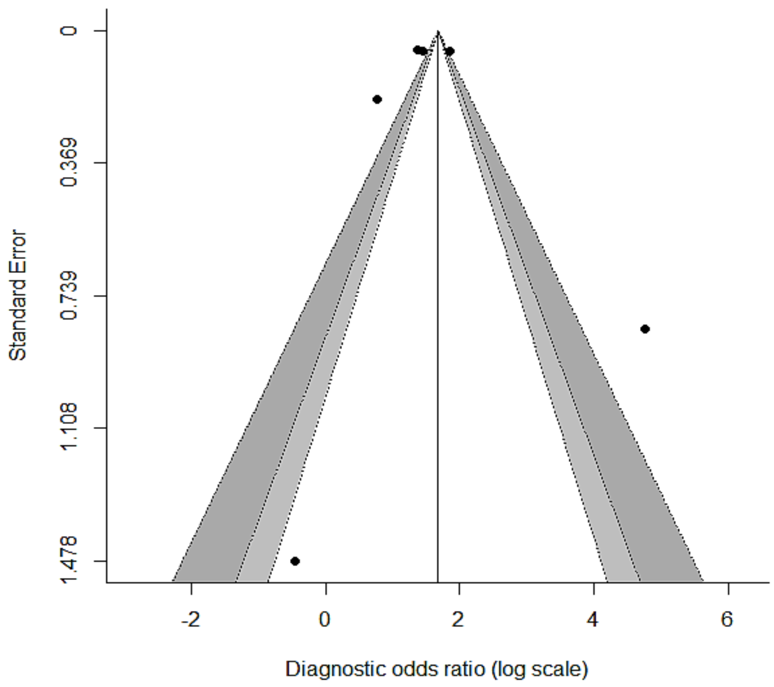

Figure S7: Funnel plot of studies evaluating predictive accuracy of age-adjusted quick sequential organ failure assessment score for mortality. No significant publication bias was detected by Egger’s test (p=0.8746).

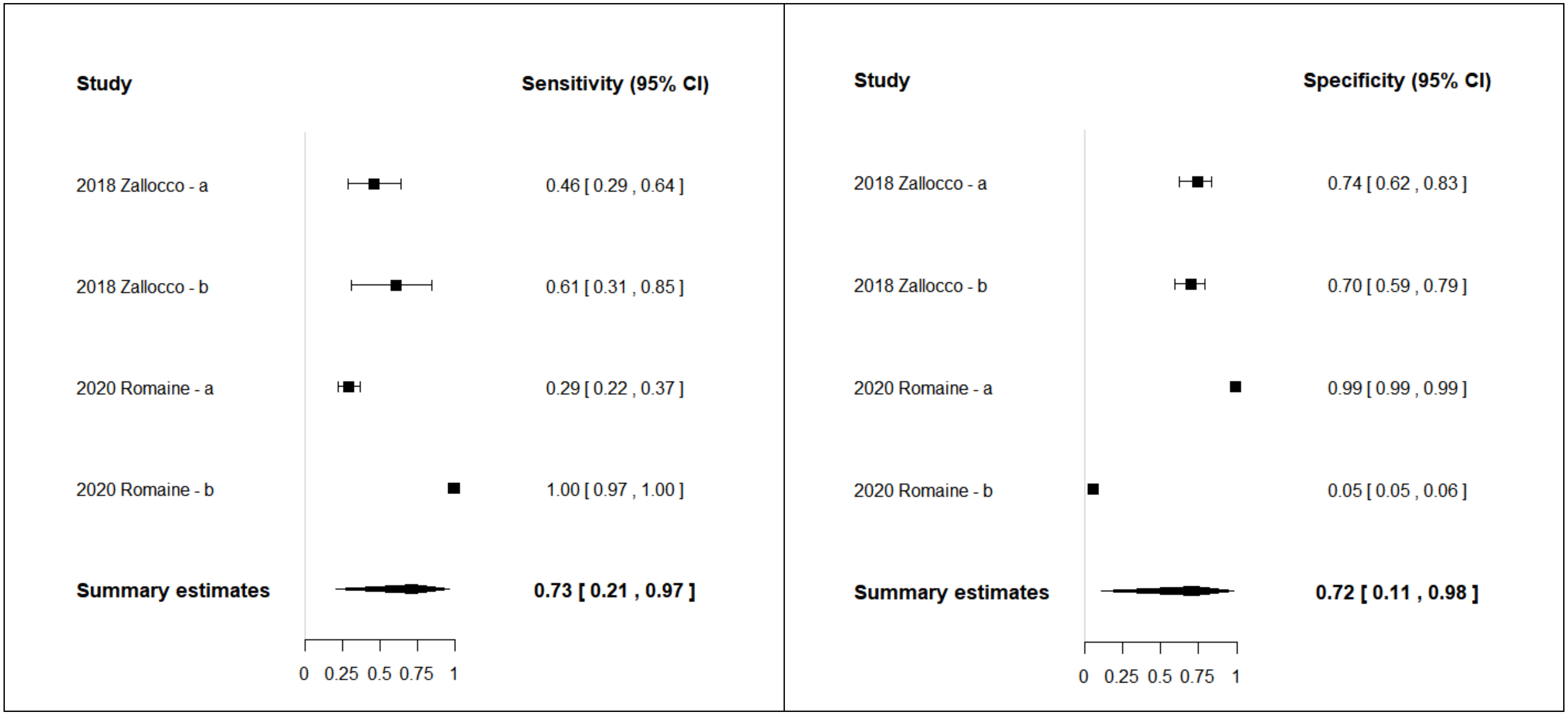

Figure S8: Coupled forest plots for sensitivity and specificity on predicting disease severity.

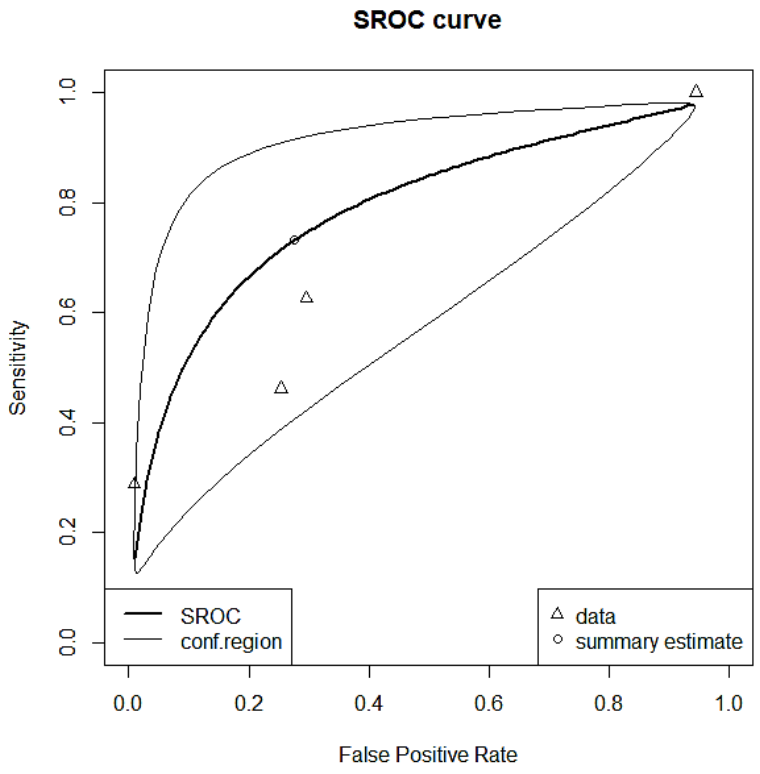

Figure S9: Summary receiver operating characteristic (SROC) curve of the predictive performance of age-adjusted quick sequential organ failure assessment score for disease severity. The area under the curve of the SROC was 0.786 (95% CI: 0.518–0.905). conf. region, 95% confidence region for SROC curve.

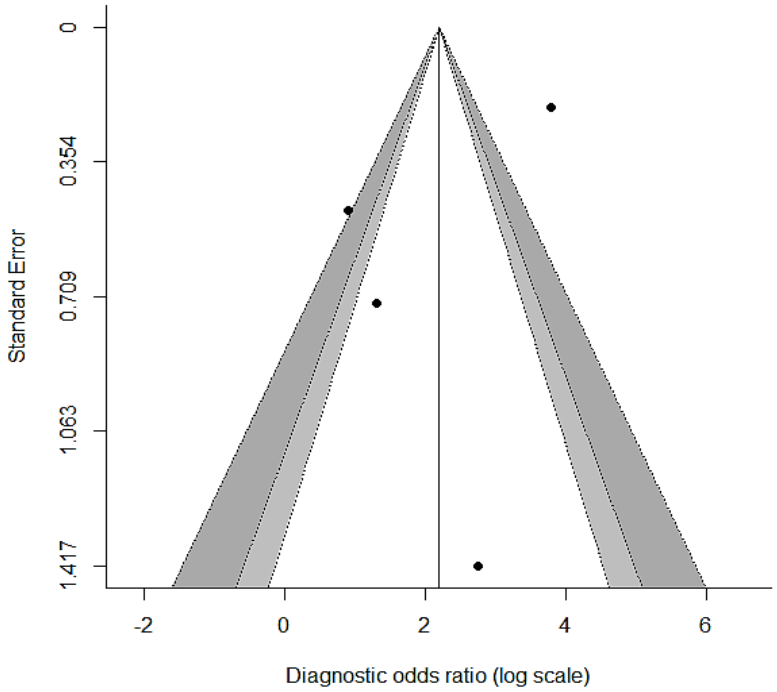

Figure S10: Funnel plot of studies evaluating predictive accuracy of age-adjusted quick sequential organ failure assessment score for disease severity. No significant publication bias was detected by Egger's test (p=0.7832).
